# Comparing the Repair of Veneered Zirconia Crowns with Ceramic or Composite Resin: An in Vitro Study

**DOI:** 10.3390/dj8020037

**Published:** 2020-04-27

**Authors:** Hattanas Kumchai, Patrapan Juntavee, Arthur F. Sun, Dan Nathanson

**Affiliations:** Department of Restorative Sciences & Biomaterials, Goldman School of Dental Medicine, Boston University, Boston, MA 02118, USA; juntavee@bu.edu (P.J.); asun@bu.edu (A.F.S.); dnathan@bu.edu (D.N.)

**Keywords:** CAD/CAM, ceramics, zirconia, prosthodontics, repair, digital dentistry

## Abstract

Statement of problem: Current techniques for repairing porcelain-chipped restorations have several limitations. With advances in CAD/CAM technology, the combination of resin cements and high-strength ceramic materials might offer new options for repairing the chipping of veneering ceramic. Purpose: The purpose of this study is to compare the load-to-failure of veneered zirconia crowns repaired by different materials. Material and Methods: Veneered zirconia crowns were made on aluminum dies (n = 10/group). Feldspathic porcelain (Vita VM9, Vident) was applied to the zirconia coping (Vita In-Ceram YZ, Vident) in a cylindrical shape (Ø 10.5 mm, height 7.5 mm). A bevel cut on the porcelain veneer (45 degree, 3 mm width) was made at one side of each crown to simulate porcelain chipping. The crowns were then divided into four different groups according to the repair materials: 1. Conventional resin composite (A; Tetric EvoCeram, Ivoclar Vivadent); 2. Flowable resin composite (B; G-aenial Universal Flo, GC america); 3. CAD/CAM milled feldspathic ceramic (C; Vita Trilux Forte, Vident); 4. CAD/CAM milled lithium disilicate glass-ceramic (D; IPS e.max CAD, Ivoclar Vivadent). Resin cement (Multilink Automix, Ivoclar Vivadent) was used to cement the CAD/CAM ceramic materials to the beveled crowns. Each crown underwent 5000 cycles of thermocycling. The strength test was performed on an Instron universal testing machine by loading force on the center of repaired part to record load-to-failure. Data were analyzed by ANOVA and Tukey HSD post-hoc tests (α = 0.05). Results: Mean loads-to-failure (in Newton +/− SD) of repaired veneered zirconia crowns were: Gr. A: 660.0 ± 200.5; Gr. B: 681.7 ± 175.9; Gr. C: 1236.0 ± 188.8; Gr. D: 1536.3 ± 286.1. Catastrophic failure was the most dominant failure mode in every group. Few specimens exhibited cohesive failure. Only one specimen in group D had adhesive failure. Conclusions: Within the limitation of the study, veneered zirconia crowns repaired with CAD/CAM ceramic materials have significantly higher load-to-failure than veneered crowns repaired with resin composite (*p* ≤ 0.05). Clinical Implications: Traditionally, porcelain-chipped restorations are often repaired with resin composite and bonding technique. Repairing chipped porcelain with CAD/CAM ceramics fitting the fractured parts can be alternative option with potential advantages. More well-designed studies are necessary to justify this novel repair technique.

## 1. Introduction

In order to improve the mechanical properties of dental prostheses, new ceramic materials and techniques have been developed and introduced in recent years. All-ceramic frameworks for fixed dental prostheses (FDP) can be made from various high-strength ceramic materials. Similar to metal-ceramics, the fabrication of zirconia based FDP uses the high strength zirconia for the framework and a veneering ceramic as the external layer. However, unlike metal ceramic restorations, there are many reports of chipping of the veneering ceramic attached to zirconia at varying rates [1,2,3,4,5].

Replacing a failed restoration with a new one is not necessarily the ideal solution. A new restoration will take additional treatment time, with potential of trauma to the tooth, plus the treatment time, and the replacement cost incurred. Some have suggested the utilization of an intraoral repair system using a resin composite. The technique uses a porcelain–resin bonding system to bond resin composite to a fractured crown. Studies have been conducted to measure the shear bond strength between the porcelain repair systems and framework materials [6,7,8]. These studies concluded that the bond strength of these two different materials could not be a permanent solution due to the lack of a sufficient bond strength. In addition, the discoloration of resin composite may be another concern. The staining of polymeric materials by food and beverages has been documented in a number of studies [9,10,11]. These substances can lead to yellow-brown stains in teeth as well as on the surfaces of the resin composites [12].

Advances in dentistry have led to the use of computer-aided design/computer-aided manufacturing (CAD/CAM) systems [13]. These machinable ceramic restorations are made from highly uniform quality crystalline content materials as compared to conventional-fabricated restorations. As a result, the clinical longevity of these restorations has also improved [14].

Resin cements continue to evolve with improved properties. With the advances in CAD/CAM technology, the combination of resin cements and high-strength ceramic materials might be one of the options for repairing of chipped veneering ceramic. However, no available studies have been found on the use of this combination as a repair technique. This study investigated various veneer porcelain repair techniques. The purpose is to compare the load-to-failure of veneered zirconia crowns repaired with different materials. The null hypothesis is that there is no difference in the load-to-failure of veneered zirconia crowns repaired with CAD/CAM ceramic materials and resin composite.

## 2. Materials and Methods

### 2.1. Experiment Overview

**Figure 1:**
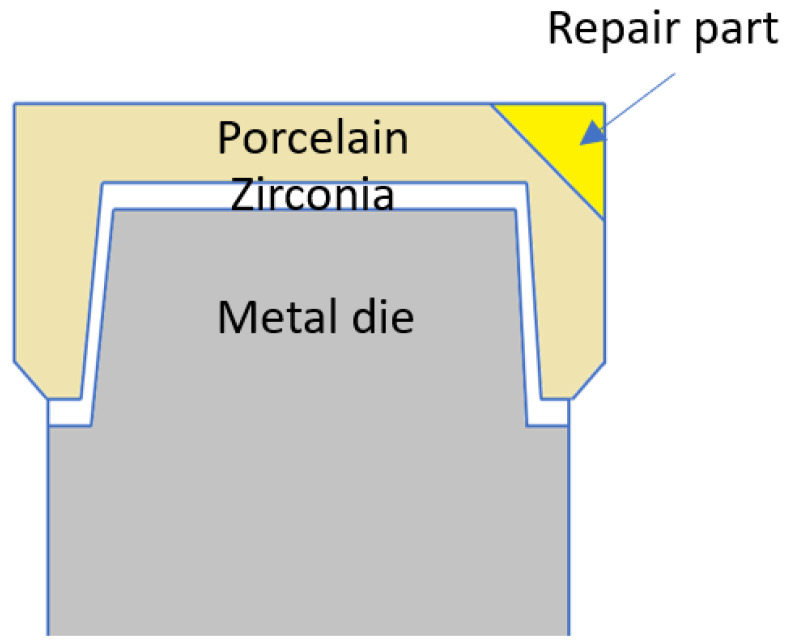
Cross sectional view of specimens used with labeled composition. Cutting plane containing the plane of symmetry of the repaired part and the cylinder axis.

**Figure 2:**
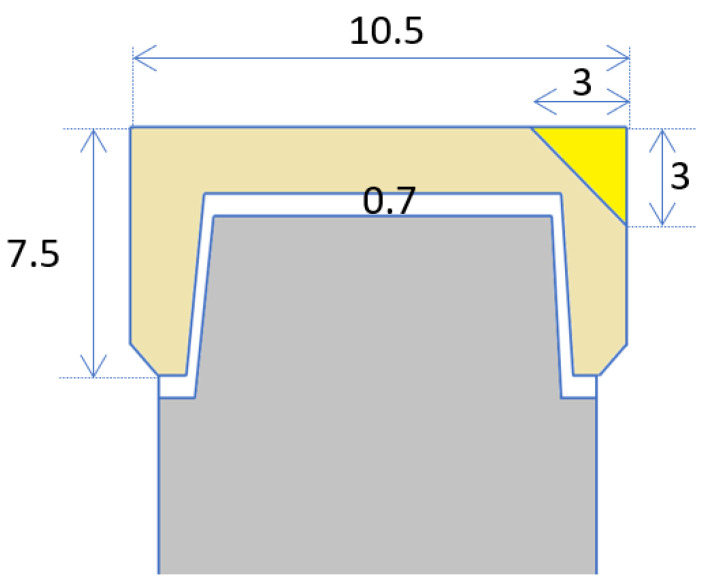
Cross sectional view of specimens with standard dimensions in millimeters. Cutting plane containing the plane of symmetry of the repaired part and the cylinder axis.

For this in-vitro study, identical zirconia specimens were made and veneered with porcelain. Specimens were prepared with identical “chips” (“artificial defects”) in the porcelain veneer, for repairs with different materials. Table 1 presents the experimental groups and the materials used in this study. Four experimental groups were assigned in this study for various materials used to repair the chipped veneered specimens (N = 40, n = 10 per group). Figure 1 and Figure 2 show the design and dimensions of the specimens.

### 2.2. Preparation of Zirconia Copings

Standardized identical aluminum dies were fabricated. One of the dies was scanned digitally with a digital laboratory scanner (inEos Blue, Sirona, Charlotte, USA) after coating the surfaces with a contrast spray (IPS Contrast Spray Labside, Ivoclar Vivadent, Schaan, Liechtenstein). Zirconia copings were designed using the software (inLab 3.88, Sirona). Die spacer thickness of 10 μm was selected. The occlusal thickness was 0.7 mm and the axial wall thickness was 0.5 mm. The copings were milled (inLab MCXL, Sirona) using pre-sintered zirconia blocks (inCoris ZI, Sirona) and then sintered (Vita Zyrcomat, Vita Zahnfabrik, Bad Säckingen, Germany) following manufacturer’s instructions as in Table 2.

### 2.3. Veneering to Zirconia Copings

Base dentin wash bake was made by mixing VM9 Base dentin powder with modeling liquid to obtain a thin aqueous mixture, applied very thinly to the zirconia coping and then fired in Vita Vacumat 6000 M furnace (Vita Zahnfabrik) following manufacturer’s instructions. Porcelain build up was completed using a Teflon mold, aluminum die, shoulder ring, and a screw. The shoulder ring was placed on the die to raise the finish line and prevent porcelain overhang during packing. The assembly (die + shoulder ring) was placed inside the Teflon mold and then In-Ceram YZ coping with the wash bake was placed on the die. Porcelain powder was mixed with modeling liquid and packed onto the mold using a vibrator. The mix was thoroughly dried using paper napkins and then the screw was used to push the die out of the mold. The specimen was carefully removed from the mold and placed on a firing tray, stabilized with fixation paste (IPS object fix, Ivoclar vivadent). Porcelain was then fired to produce over-sized crowns following manufacturer’s instructions as in Table 3.

### 2.4. Cementing of Over-Sized Crowns to Aluminum Dies

Before cementing, the internal surfaces of all crowns were sandblasted with 50 μm Al_2_O_3_ (Cobra Aluoxid rosa, Renfert GmbH-Industriegebiet Hilzingen, Hilzingen, Germany) at 2 bar pressure for 5 s, cleaned in ultrasonic cleaner for 5 min then dried with compressed oil-free air for 20 s. ‘Monobond plus’ was applied to the pre-treated surface with a brush and allowed to react for 60 s. Subsequently it was dispersed with a strong stream of air. Multilink primers A and B were mixed at 1:1 ratio and applied to the surface of aluminum dies for 15 s. A thin layer of luting composite (Multilink Automix, Ivoclar Vivadent) was applied on the internal surfaces of all crowns and then seated on the dies with finger pressure and excess cement was cleaned using a disposable brush. Specimens were then placed in a cementing apparatus under 1.85 kg load for 10 min. Light-curing was conducted by pre-cure for 1 s before removal of cement excess, and then a full-cure for 20 s.

### 2.5. Finishing of Cemented Crowns

The crowns were then machined into standardized cylindrical shape with a CNC lathe machine (Compact 5, EMCO Maier, Taxach, Austria) and installed in custom holders for linear precision saw machine (Isomet 2000, Buehler, Lake Bluff, USA). The crowns were finished using fine diamond burs and polishing rubber wheels then glazed using Akzent glaze powder and liquid with manufacturer’s instruction.

### 2.6. Simulated Chipping of Cemented Crowns

**Figure 3:**
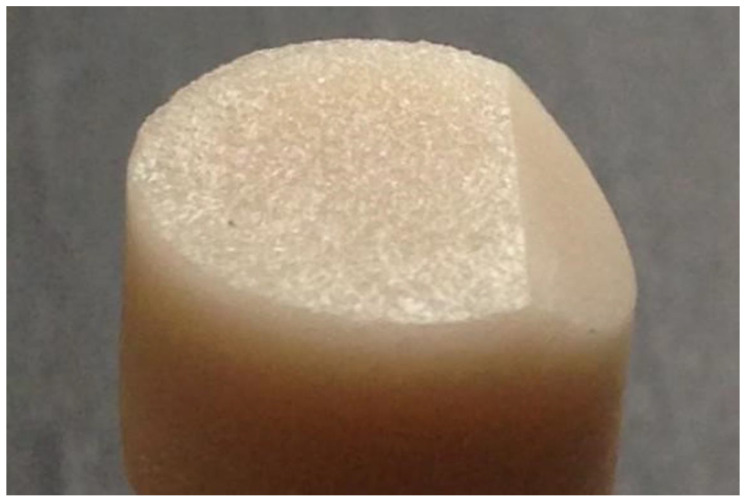
Beveled crown.

Cemented crowns were put in a customized stub. This stub allowed the crowns to be placed at 45 degrees to the wafering blade of the Isomet 2000 Precision saw (Buehler, USA). A bevel-cut on the porcelain veneer (45 degree, 3 mm width) was made on one side of each crown with 15LC diamond-wafering blade mounted on an Isomet 2000 Precision Saw. The cuts were made at 800 rpm with 300 g of load with cooling provided by a dual-nozzle water irrigation system. Figure 3 shows the crown after beveling.

### 2.7. Repair of Chipped Veneered Crowns

The bevel-area of each porcelain ‘defect’ was etched with 5% Hydrofluoric acid (IPS Ceramic Etching Gel, Ivoclar Vivadent) for 1 min as recommended by the manufacturer. After etching, the specimens were thoroughly rinsed for 30 s using an air water spray to ensure that all the acid was removed from the surface. A silanating agent (Monobond-Plus, Ivoclar Vivadent) was applied to the surface of the “defect” for 60 s and dried it with an air syringe. The crowns were subsequently repaired according to the assigned groups:

Group A: A custom clear shell was made to capture the shape of the intact crown before simulated chipping was performed. It was made from vacuum forming materials (Clear mouth guard material 1 mm, Great lakes orthodontics). The material was heated until the sag was formed and adapted to the specimen by using vacuum former machine (Keystone dental). The excess material was removed afterwards with scissor. A small hole was made with a round diamond bur #2 (Brasseler, Savannah, USA) to provide venting for the resin composite. A thin layer of Heliobond (Ivoclar Vivadent) was applied onto porcelain defect using a brush. An optimal, thin layer can be achieved using a stream of air. The area was light-polymerized for 10 s with a visible light curing unit (Blue phase, Ivoclar Vivadent). Resin composite (Tetric EvoCeram, Ivoclar Vivadent) was applied onto the defect with the help of custom clear-shell in order to control the shape. The area was then light-polymerized for 40 s with a visible light curing unit (Blue phase, Ivoclar Vivadent). The shell was removed afterward. The area was polished with rubber wheel.

Group B: A thin layer of Self-etch bonding agent (G-aenial bond, GC America, Alsip, USA) was applied onto porcelain defect using a brush. An optimal, thin layer can be achieved using a stream of air. The area was light-polymerized for 10 s with a visible light curing unit (Blue phase, Ivoclar Vivadent). Resin composite (G-aenial Evo Flo, GC America) was injected onto the defect through a hole of the same custom clear-shell previously mentioned in group A. The area was then light-polymerized for 40 s with a visible light curing unit (Blue phase, Ivoclar Vivadent). The shell was removed afterward. The area was polished with rubber wheel.

Group C: Three master patterns of repair part were fabricated with Inlay pattern resin (Duralay, Reliance dental mfg). The monomer and powder were mixed together and adapted to the porcelain defect. The clear-shell from group A was adapted onto the crown in order to obtain the shape of the repair parts. Three patterns were connected together using sprues and then scanned digitally with scanning machine (inEos Blue, Sirona) after coating the surfaces with a contrast spray (IPS Contrast Spray Labside, Ivoclar Vivadent). The repair parts were milled using feldspathic ceramic blocks (Vita Trilux Forte, Vita Zahnfabrik). The spruces were cut after the milling. The occlusal surface of the parts was polished with rubber wheel and glazed using Akzent glaze powder and liquid. The repair parts were etched with 5% Hydrofluoric acid (IPS Ceramic Etching Gel, Ivoclar Vivadent) for 1 min. Silanating agent (Monobond-Plus, Ivoclar Vivadent) was applied on the inner surface of repair parts for 60 s and dried with an air syringe. The repair parts were cemented to the crowns with resin cement (Multilink Automix, Ivoclar Vivadent). The area was then light-polymerized for 40 s with a light curing unit (Blue phase, Ivoclar Vivadent).

**Figure 4:**
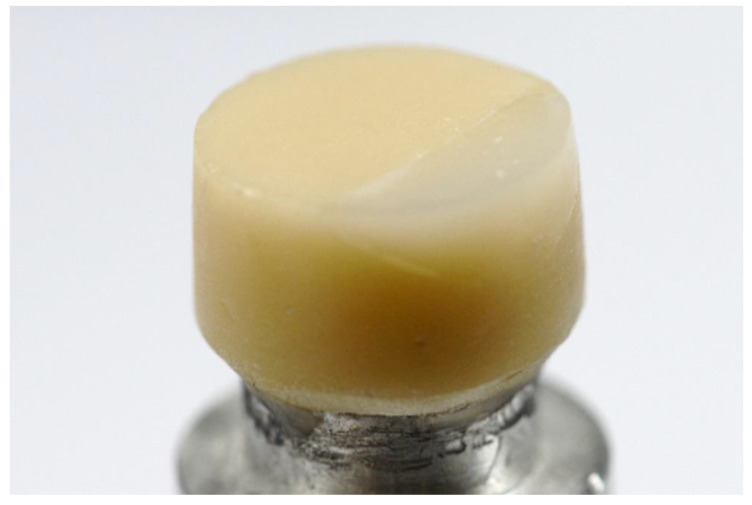
Repaired specimen with bonded ceramic.

Group D: The repair parts were milled using IPS e.max CAD (Ivoclar Vivadent). The spurs were cut after the milling. The occlusal surface of the parts was polished with rubber wheel at the occlusal surface. The parts were then subjected to crystallization firing using Programat EP5000 furnace (Ivoclar vivadent) and glazed on the occlusal surface simultaneously using IPS e.max CAD Crystall glaze paste (Ivoclar Vivadent). The cementing of the repaired parts to the crowns was done as previously mentioned in group C. Figure 4 shows specimens after repairing with bonded ceramic.

### 2.8. Thermal Cyclic

All crowns were subjected to thermal cycling for 5000 cycles in water baths held at 5 °C and 55 °C, with a dwell time in each bath of 30 s and a transfer time of 20 s. After simulated aging, the specimens were subsequently subjected to the mechanical testing.

### 2.9. Testing

Mechanical testing was performed on Instron 5566A Universal Testing Frame (Intron, Norwood, MA, USA) with 10kN load cell by loading force on the center of repaired part to record the load-to-failure. The force was applied through a stainless-steel ball (2.5 mm in diameter). The load was applied vertically and colinear from the crown axis to the center of the repaired part at a crosshead speed of 0.5 mm/min. The fracture load was registered as a peak in the load-displacement tracing, recorded in newtons (N). The mode of fracture was examined for each specimen and categorized according to the following descriptions; type I fracture of the repair part without damaging of the porcelain (cohesive failure), type II fracture of the repair part with the porcelain (catastrophic failure), and type III de-bonding of the repair part from porcelain (adhesive failure).

### 2.10. Scanning Electron Microscope (SEM)

Sectioned specimens were glued on aluminum stubs (SPI supplies, Boston, USA). Specimens were silver painted on the sides and allowed to dry for 24 h. The specimens then were sputter coated with gold/palladium using sputter coater (Hummer II Technics). The specimens were viewed under scanning electron microscope SEM (PHILIPS XL 30 SEM, Phillips Electronics, Cambridge, USA) with an acceleration voltage of 15 kV to evaluate the fracture surfaces.

### 2.11. Statistical Analysis

Statistical analysis was performed with SPSS Statistics for Windows v.11 (SPSS). Data were analyzed using one-way ANOVA, and multiple comparisons were made using Tukey HSD post hoc test. The *p* value < 0.05 was considered to be statistically significant in all tests.

## 3. Results

The means and standard deviations for load-to-failure of repaired veneered zirconia crowns are presented in Table 4. ANOVA test (Table 5) revealed that there was a statistically significant difference between the groups (*p* < 0.05). The Tukey HSD test indicated that there was a significantly higher (*p* < 0.05) mean load-to-failure value for group D (1536.3 ± 286.1 N) when compared to group A, B, and C (660.0 ± 200.5 N, 681.7 ± 175.9 N, and 1236.0 ± 188.8 N, respectively). Group C had significantly higher mean load-to-failure when compared to group A and B. There was no significant difference in fracture resistance between groups A and B.

Modes of failure of the three tested CAD/CAM blocks are presented in Table 6. The results showed that catastrophic failures were the most dominant failure modes in every group. A number of specimens exhibited cohesive failure. Only one specimen in group D had adhesive failure.

### Scanning Electron Microscope Evaluation

**Figure 5:**
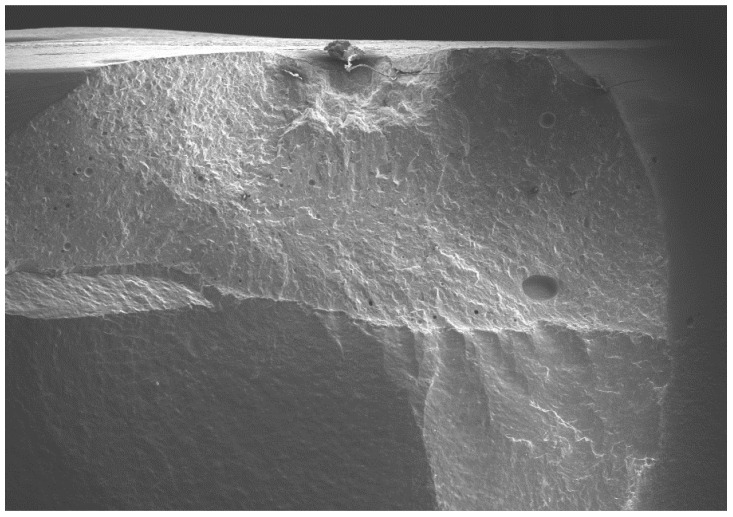
SEM micrograph of the fractured veneered-zirconia crown repaired with Tetric EvoCeram at magnification of 22×.

**Figure 6:**
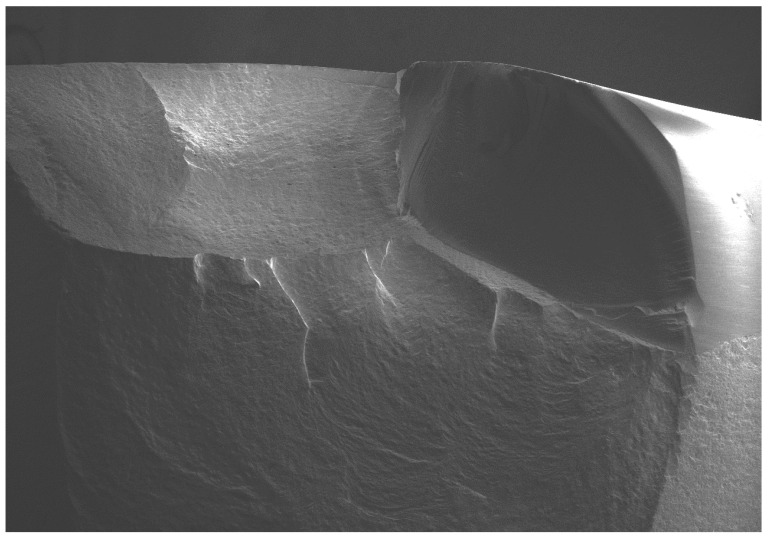
SEM micrograph of the fractured veneered-zirconia crown repaired with G-aenial Universal Flo at magnification of 22×.

**Figure 7:**
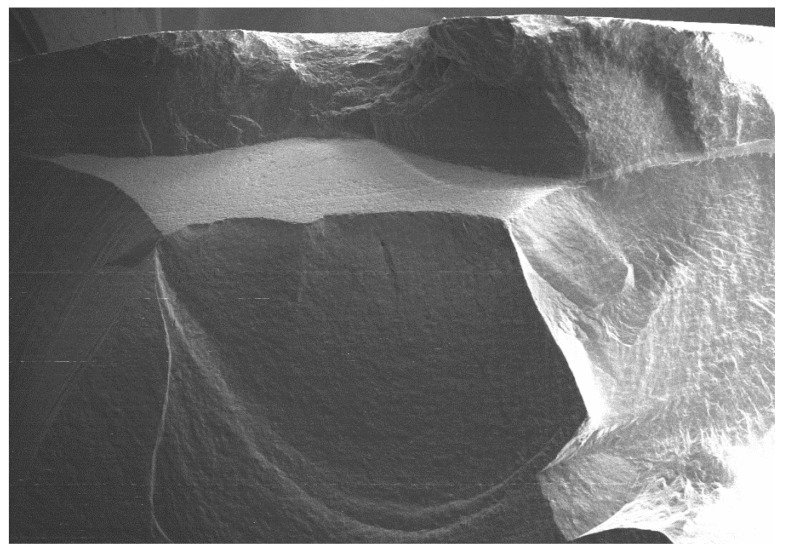
SEM micrograph of the fractured veneered-zirconia crown repaired with Vita Triluxe Forte at magnification of 22×.

**Figure 8:**
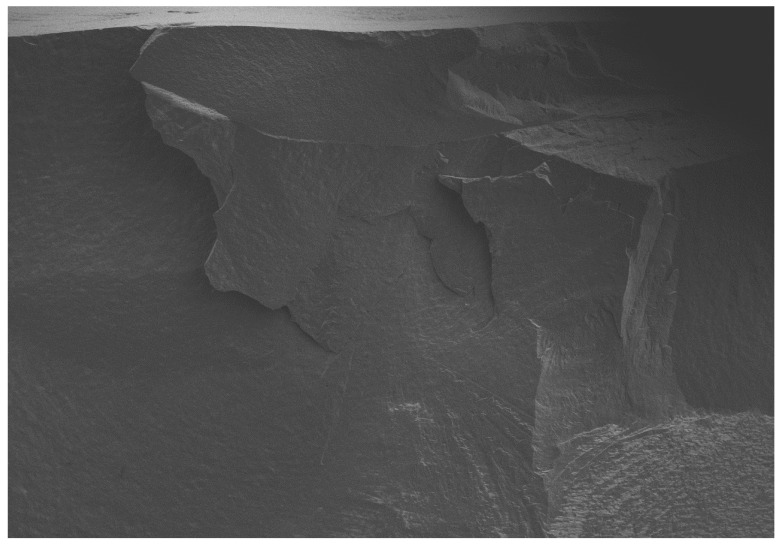
SEM micrograph of the fractured veneered-zirconia crown repaired with IPS e.max CAD at magnification of 22×.

All of the selected specimens demonstrated catastrophic failures (Figure 5, Figure 6, Figure 7 and Figure 8). They all exhibited similar fracture patterns. The fracture origin was from the top of the contacting point between ball load and specimens. The crack then propagated in a downward direction.

## 4. Discussion

The crown repair system detailed in this study was assessed in-vitro by mechanical testing to find the levels of resistance of the four tested groups, by subjecting them to loading-to-failure. The idea was to simulate resistance up to clinical failure. Compressive load at failure (i.e., by a mechanical instrument) is a measurement of the load at which a specimen shows the first sign of failure under test conditions.

To standardize the experimental methods and conditions, multiple factors must be defined and controlled. The metal dies used in this study were industrially manufactured with dimensions according to the clinical guideline. The zirconia copings and the repair parts were standardized with respect to the design, shape, size, and thickness. Beveling procedures to simulate porcelain chipping were accomplished using an Isomet 2000 Precision saw (Buehler) with a custom jig to ensure accuracy. Beveling at 45 degrees was used, as this caused the highest shear stress to the specimens during vertical loading.

The surface treatment of the porcelain was identical for all the groups. The porcelain was etched with 5% Hydrofluoric acid and treated with silane coupling agent. This is the preferred surface pretreatment technique for achieving high bond strength for silica-based all-ceramic restorations [15]. The hydrofluoric acid etched the surface of the porcelain to create micro-porosities in order to achieve sufficient activation to facilitate micromechanical and chemical bonding between the ceramic material and the resin material [2,8,16]. Silane coupling agents promote adhesion and form a chemical bond with organic and inorganic surfaces, thereby increasing the wettability of the ceramic surfaces [17].

Due to the limitation of CAD software in designing a non-anatomic structure, CAD/CAM repair parts in this study could not be designed directly from the software. The digital design of the CAD/CAM repair parts were from the inlay pattern resin instead. Despite this additional step, there was no difference in the final specimens’ shapes and texture as the specimens were polished and the dimensions were controlled afterward.

When the mean fracture resistance of the four groups was compared, group C and D had higher fracture resistance values than groups A and B. The higher strength values of groups C and D could be attributed to the repair materials and the resin cement. Both CAD/CAM feldspathic ceramic and lithium disilicate glass-ceramic had higher flexural strength than resin composite [18,19]. CAD/CAM materials were made using standardized manufacturing processes with uniform material quality. The resin cement used in this study might have also contributed to the results. Another study had shown that the shear bond strength of porcelain veneer to resin cement was higher than to flowable resin composite [20].

Lithium disilicate glass-ceramic was shown to have a flexural strength of approximately 360 MPa while feldspathic ceramic had flexural strength of 130 MPa [18,19]. There was no difference in fracture resistance values between group A and B. Both groups had resin composite as the repair materials. Both of the resin composites used in this study were considered nano-hybrid composite.

In-vitro evaluation is used for testing any new material or technique to examine the properties and potential that it possesses. Because our study only tested the load-to-failure of the repaired crowns in vertical loading, it is suggested that other aspects of the test, such as the effect of repair size, loading angulation, thermal cycling, cyclic fatigue, mode of failure and micro leakage should be studied for a more comprehensive evaluation of these repair systems.

## 5. Conclusions

Within the limitation of this study, the following observations were made:

- Veneered zirconia crowns repaired with CAD/CAM ceramic materials had significantly higher load-to-failure than veneered crowns repaired with resin composite.
- Veneered zirconia crowns repaired with CAD/CAM-milled lithium disilicate glass-ceramic had significantly higher load-to-failure than veneered crowns repaired with CAD/CAM-milled feldspathic ceramic.
- There was no difference in load-to-failure between veneered zirconia crowns repaired with conventional resin composite or flowable resin composite.

## Figures and Tables

**Table 1:** Experimental groups and materials used in this study.

Group	Repair Materials	Bonding Agents	Resin Cement
A	Conventional resin composite (Tetric EvoCeram, Ivoclar Vivadent)	Total-etch bonding agent (Heliobond, Ivoclar Vivadent)	-
B	Flowable resin composite (G-aenial Universal Flo, GC america)	Self-etch bonding agent (G-aenial bond, GC america)	-
C	CAD/CAM feldspathic ceramic (Vita Trilux Forte, Vita Zahnfabrik)	-	Dual cure resin cement (Multilink Automix, Ivoclar Vivadent)
D	CAD/CAM lithium disilicate glass-ceramic (IPS e.max CAD, Ivoclar Vivadent)	-	Dual cure resin cement (Multilink Automix, Ivoclar Vivadent)

**Table 2:** Firing parameter for zirconia sintering.

Materials	Entry Temperature	Rising Temperature Rate (°C/min)	Final Temperature (°C)	Holding Time (min)
inCoris ZI	Room temperature	17	1500	120

**Table 3:** Firing steps for Vita VM9.

Firing Steps	Drying Temp. °C	Drying Time min.	Rising Time min.	Rising Rate °C/min	Final Temp °C	Holding Time min.	Cooling Temp. °C	Vacuum min.
Wash bake firing	500	2.00	7.27	45	950	1.00	-	8.11
1st dentin firing	500	6.00	7.27	45	910	1.00	600	7.27
2nd dentin firing	500	6.00	7.16	45	900	1.00	600	7.16
Glaze firing	500	0.00	5.00	45	900	1.00	600	-

**Table 4:** Means and S.D. (N) for load-to-failure of repaired veneered zirconia crowns. Same lower-case letters in each column indicate no significant differences (*p* > 0.05).

Group	Load-To-Failure (N)		
	Average	Standard Deviation	Coefficient of Variation
A	660.0 ^c^	200.5	30.3
B	681.7 ^c^	175.9	25.8
C	1236.0 ^b^	188.8	15.2
D	1536.3 ^a^	286.1	18.6

**Table 5:** One-way ANOVA test, failure load of repaired veneered zirconia crowns.

Source	DF	Sum of Squares	Mean Square	F Ratio	Prob > F
Group	3	5,691,447.6	1,897,149	38.1603	<0.0001
Error	36	1,789,748.6	49,715		
C. Total	39	7,481,196.2			

**Table 6:** Mode of failure of repaired veneered zirconia crowns.

Group	Failure Mode (n)		
	Cohesive Failure	Catastrophic Failure	Adhesive Failure
A	3	7	0
B	1	9	0
C	2	8	0
D	2	7	1
